# Supplementary material for: The Anti-ROR1 Monoclonal Antibody Zilovertamab Inhibits the Proliferation of Ovarian and Endometrial Cancer Cells
Source: Pharmaceutics. 2022 Apr 11;14(4):837. doi: 10.3390/pharmaceutics14040837 (PMC9033071; doi:10.3390/pharmaceutics14040837)
Supplement: Supplementary file 1 [file pharmaceutics-14-00837-s001.zip › pharmaceutics-1641991-supplementary.pdf]

pharmaceutics

MDPI

Supplementary Materials: The Anti-ROR1 Monoclonal Antibody Zilovertamab Inhibits the Proliferation of Ovarian and Endometrial Cancer Cells

Dongli Liu, Gunnar F. Kaufmann, James B. Breitmeyer, Kristie-Ann Dickson, Deborah J. Marsh and Caroline E. Ford

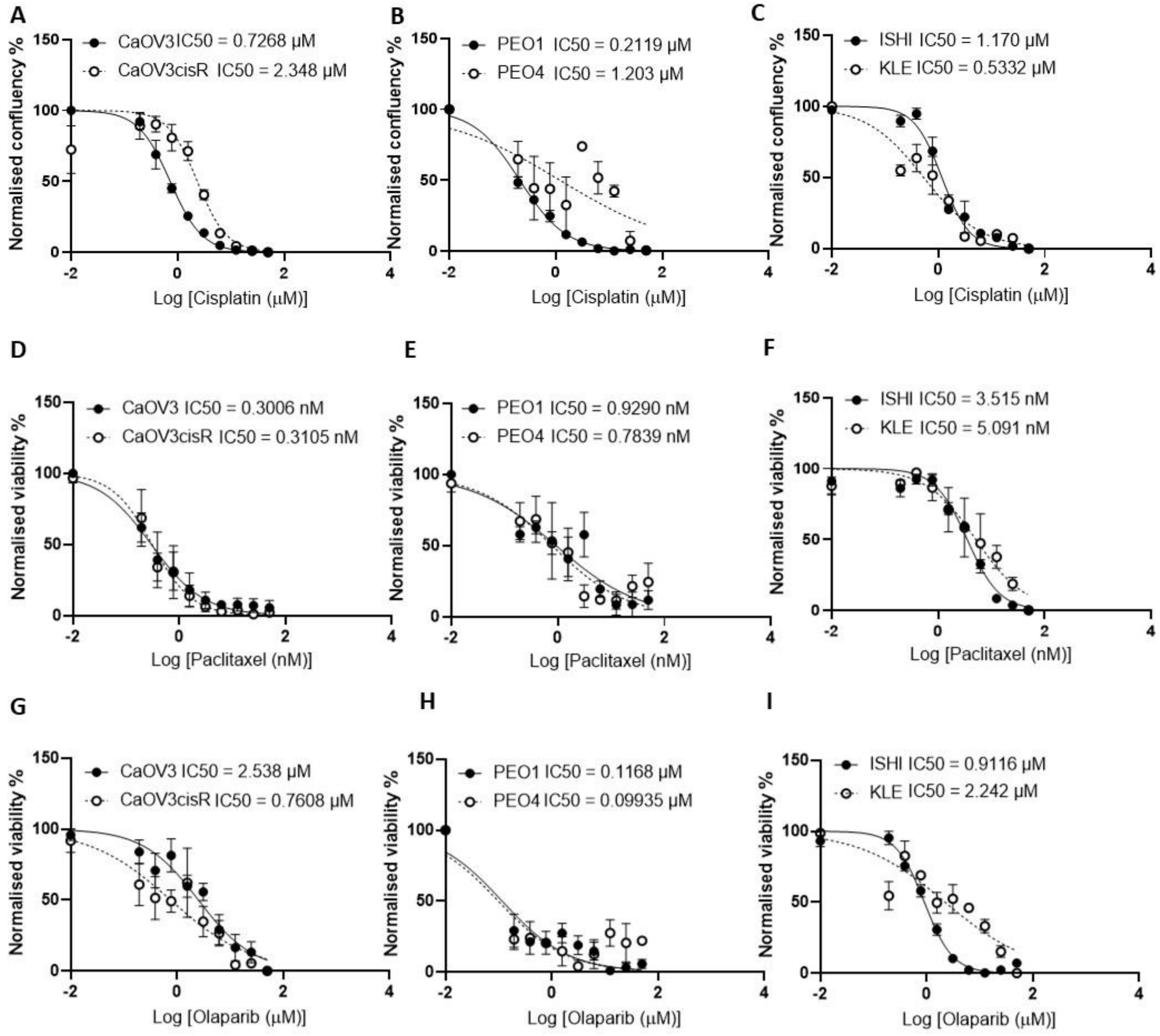

Figure S1. Dose response curves for the chemotherapy agents in ovarian and endometrial cancer cell lines (CaOV3, CaOV3CisR, PEO1, PEO4, Ishikawa and KLE) with half maximal inhibitory concentration (IC50) at 72 h post treatment. (**A**-**C**). Dose response curves of cisplatin. (**D**-**F**). Dose response curves of paclitaxel. (**G**–**I**). Dose response curves of olaparib. For each panel, $n = 3$, error bar SEM.

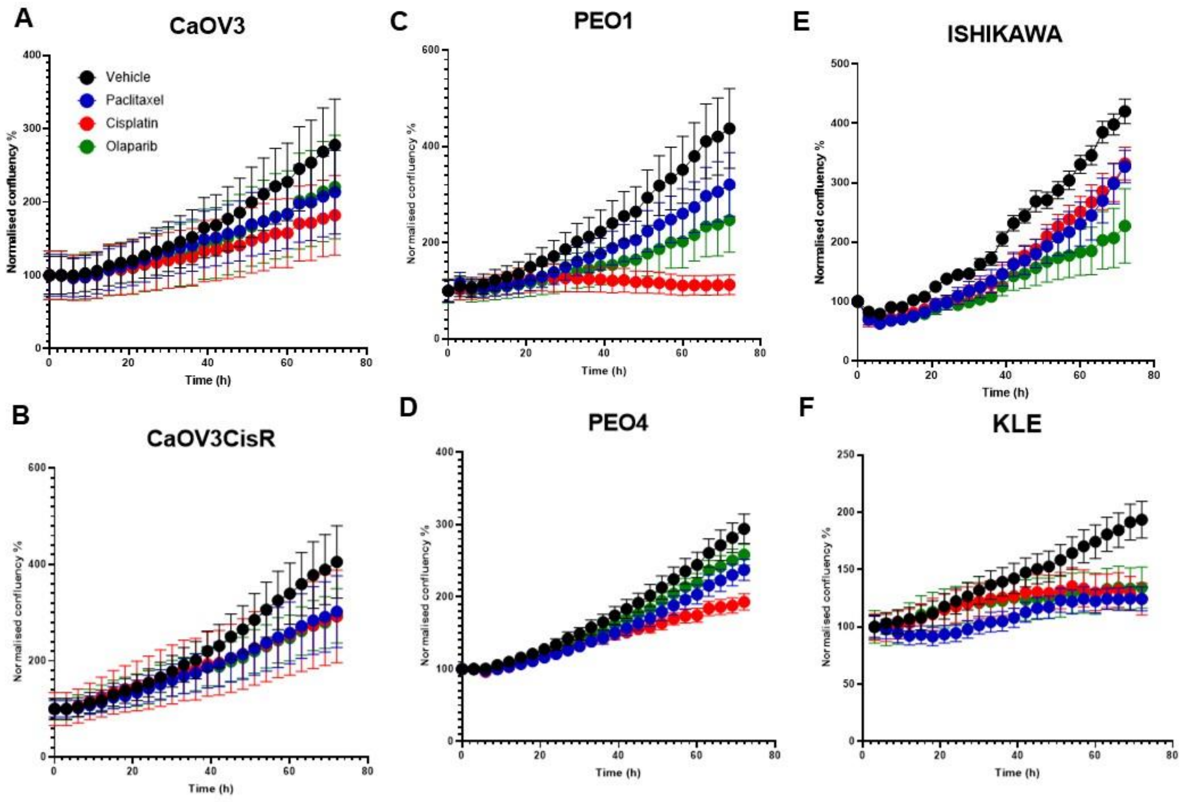

Figure S2. Cell confluence analysed via the IncuCyte S3 live imaging system over a period of 72 h. Compared to vehicle control, treating high grade ovarian cancer cell lines CaOV3 (**A**), CaOV3CisR (**B**), PEO1 (**C**), PEO4 (**D**) as well as endometrial cancer cell lines Ishikawa (**E**) and KLE (**F**)cells with paclitaxel, cisplatin or olaparib at IC70 dose significantly inhibited cell proliferation at 72 h.

Table S1. Primer sequences used for qRT-PCR.

	**Forward (5′-3′)**	**Reverse (5′-3′)**
ROR1	CAACAAGAAGCCTCCCTAATGG	CCTGAGTGACGGCACCTAGAA
RHOA	GGAAAGCAGGTAGAGTTGGCT	GGCTGTCGATGGAAAAACACAT
VIM	CCAAACTTTTCCTCCCTGAACC	GTGATGCTGAGAAGTTTCGTTGA
CDH1	TGAGTGTCCCCCGGTATCTTC	CAGTATCAGCCGCTTTCAGATTTT
SDHA	TGGGAACAAGAGGGCATCTG	CCACCACTGCATCAAATTCATG
HSPCB	TCTGGGTATCGGAAAGCAAGCC	GTGCACTTCCTCAGGCATCTTG
RPL13a	CCTGGAGGAGAAGAGGAAAGAGA	TTGAGGACCTCTGTGTATTTGTCAA

Table S2. Cell confluence differences following different treatment conditions measured by IncuCyte S3 platform in the high grade serous ovarian cancer and endometrial cancer cell lines. Two-way ANOVA was performed with a Tukey correction for multiple t testing. Data presented Mean differences of cell confluency (adjusted *p*) at 72 h. *padj < 0.05. **padj < 0.01. ***padj < 0.001. Statistical significance was cut off at 0.05 and highlighted with shading.

	CaOV3	CaOV3CisR	PEO1	PEO4	Ishikawa	KLE
**Vehicle vs. Zilo (25)**	21.480 (<0.001***)	21.420 (0.011*)	26.200 (<0.001***)	5.985 (<0.001***)	24.530 (<0.001***)	11.250 (0.078)
**Vehicle vs. Zilo (50)**	37.160 (<0.001***)	32.100 (<0.001***)	30.000 (<0.001***)	5.955 (<0.001***)	60.040 (<0.001***)	16.090 (0.004**)
**Paclitaxel vs. Zilo (25) + Pac**	−2.520 (0.913)	13.010 (0.306)	8.338 (0.343)	3.087 (0.140)	4.987 (0.703)	1.893 (0.886)
**Zilo (25) vs. Zilo (25) + Pac**	−12.44 (0.112)	3.144 (0.933)	1.337 (0.973)	−1.806 (0.507)	2.788 (0.896)	11.510 (0.013*)
**Paclitaxel vs. Zilo (50) + Pac**	25.590 (<0.001***)	20.870 (0.001**)	16.620 (0.017*)	6.485 (<0.001***)	35.480 (<0.001***)	6.959 (0.332)
**Zilo (50) vs. Zilo (50) + Pac**	−0.2712 (0.997)	0.319 (0.998)	5.824 (0.598)	1.621 (0.514)	−2.227 (0.799)	11.730 (0.045*)
**Cisplatin vs. Zilo (25) + Cis**	−1.234 (0.805)	14.67 (0.166)	1.042 (0.975)	1.059 (0.766)	8.809 (0.254)	−1.851 (0.934)
**Zilo (25) vs. Zilo (25) + Cis**	−4.974 (0.682)	11.05 (0.359)	27.8 (<0.001***)	0.190 (0.991)	1.459 (0.963)	8.014 (0.284)
**Cisplatin vs. Zilo (50) + Cis**	19.350 (<0.001***)	16.070 (0.041*)	4.117 (0.676)	4.318 (0.005**)	31.03 (<0.001***)	6.457 (0.475)
**Zilo (50) vs. Zilo (50) + Cis**	−0.061 (0.999)	1.767 (0.951)	27.08 (<0.001***)	3.479 (0.033*)	−11.83 (0.021*)	11.480 (0.098)
**Olaparib vs. Zilo (25) + Ola**	2.913 (0.892)	13.970 (0.116)	4.813 (0.770)	−1.108 (0.701)	−15.050 (0.079)	−5.115 (0.526)
**Zilo (25) vs. Zilo (25) + Ola**	−2.985 (0.887)	11.33 (0.241)	9.292 (0.380)	−3.564 (0.028*)	−8.473 (0.442)	11.17 (0.049*)
**Olaparib vs. Zilo (50) + Ola**	19.440 (<0.001***)	18.10 (<0.001***)	9.048 (0.349)	1.367 (0.599)	21.420 (<0.001***)	−3.480 (0.794)
**Zilo (50) vs. Zilo (50) + Ola**	−2.136 (0.872)	4.775 0.417	9.729 (0.296)	−1.060 (0.734)	−7.517 (0.306)	7.960 (0.302)
